# Oral l-Cysteine Supplementation Enhances the Long Term-Effect of Topical Basic Fibroblast Growth Factor (bFGF) in Reducing the Corneal Haze after Photorefractive Keratectomy in Myopic Patients

**DOI:** 10.3390/ph13040067

**Published:** 2020-04-15

**Authors:** Alessandro Meduri, Loredana Bergandi, Pietro Perroni, Francesca Silvagno, Pasquale Aragona

**Affiliations:** 1 Biomedical, Dental and Morphological and Functional Images Sciences, Department, University of Messina, 98122 Messina, Italy; ameduri@unime.it (A.M.); pie_89_@hotmail.it (P.P.); paragona@unime.it (P.A.); 2 Department of Oncology, University of Torino, 10126 Torino, Italy; francesca.silvagno@unito.it

**Keywords:** photorefractive keratectomy, corneal haze, basic fibroblast growth factor, l-cysteine administration, corneal re-epithelialization, corneal haze

## Abstract

We aimed at evaluating the long-term effects of l-cysteine oral supplementation to basic fibroblast growth factor (bFGF) eye-drops on corneal re-epithelization and transparency in myopic patients subjected to photorefractive keratectomy (PRK). Forty patients subjected to bilateral PRK for myopia were enrolled and randomly divided into two groups receiving an additional therapy together with the standard postoperative treatment consisting in local tobramycin 0.3%, dexamethasone 0.1%, diclofenac 0.1%, and 0.2% hyaluronate. Group 1 included 20 patients (11 males and 9 females; 34.09 ± 8 years of age) receiving only bFGF eye-drops (10 μg/10 μL) four times a day for 7 days starting from the day of surgery; Group 2 included 20 patients (12 males and 8 females; 37.35 ± 11.5 years of age) who were postoperatively administered with topical basic fibroblast growth factor (bFGF; 10 μg/10 μL) four times a day for 7 days plus oral l-cysteine supplementation (500 mg/capsule) once a day for 15 days, starting 7 days before PRK. Patients were followed-up for 12 months. Clinical ophthalmologic parameters were recorded for all the 80 examined eyes. The corneal transparency was evaluated in vivo by slit lamp and confocal microscopy. The data showed that: (a) the corneal haze occurred in a smaller percentage of the patients who were postoperatively administered with topical bFGF plus oral l-cysteine supplementation (Group 2) compared to patients who received only bFGF (Group 1); (b) at 6 months of follow-up, the stromal mean image brightness of the patients belonging to Group 2 was significantly lower than that of the Group 1 (*p* < 0.03), and, interestingly, the difference was even more evident at 12 month from the treatment (*p* < 0.001). Moreover, the final mean of the spherical equivalent refraction was −0.06 ± 0.2 D in Group 1 and −0.08 ± 0.3 D in Group 2, whereas the final uncorrected distance visual acuity (UDVA) was equal or superior to 20/25 in 100% of eyes in both Group 1 and 2. Post refractive patients can benefit from the administration of l-cysteine before the surgery and in association with bFGF in the early postoperative period, showing a faster corneal re-epithelization able to prevent corneal haze in the long-term recovery.

## 1. Introduction

Corneal wound healing complications associated with photorefractive keratectomy (PRK) include, mainly, haze onset and refractive regression [1].

The PRK surgery entails the laser-assisted removal of the epithelium with underlying Bowman’s layer, the basement membrane and variable part of the anterior stroma, depending on the degree of correction needed [1]. The initial epithelial coverage occurs fairly quickly, but the complete restoration of epithelial basement membrane, the corneal nerve regeneration, and the stromal remodeling usually take months to years [2,3]. In order to achieve the corneal transparency necessary to restore the corneal function and, thus, a proper visual quality after refractive surgery, a correct and rapid recovery of the superficial epithelial layer is of paramount importance to guarantee a complete corneal regeneration [4]. Therefore, it is clear that a quicker and better healing process is an important contributor to the outcome that is a critical point for a successful PRK surgery whereas a prolonged epithelial wound healing is one of the main PRK complications [5].

Corneal wound healing is a complex process regulated by the interaction between epithelial and stromal cells, nerve fibers, and the tear film [6]. Several studies have confirmed the role of epithelial cells in the release of growth factors, in addition to cytokines and matrix metalloproteases, necessary for the corneal regeneration process after PRK [1,2,5,7,8]. As a consequence of the epithelial damage and of the loss of the intact basement membrane function, also stromal cells and keratocytes secrete cytokines in order to modulate the proliferation, migration and differentiation of epithelial and stromal cells into fibroblasts and myofibroblasts [3,9] and to repair the stroma [6].

In rabbit models, the topical application of a variety of molecules is able to promote the corneal repair process leading to a better wound healing; among the tested factors, can be mentioned the plasma rich in growth factors (PRGF-Endoret) [10], the ascorbic acid [11], the nerve growth factor (NGF) in combination with docosahexaenoic acid (DHA) [12], the vitamin E and hydrocortisone acetate [13], and finally the basic fibroblast growth factor (bFGF) [14,15] can be mentioned. Moreover we previously showed that, in transgenic mice, topical cytochrome c peroxidase [16], bFGF alone [17], or in combination with cytochrome c [18] and oral administration of l-cysteine [19] significantly accelerates epithelial healing after excimer photoablation. Despite the limits of the animal models, which are the different physiology and the short term evaluation, we found that these observations were a useful starting point to test the efficacy of supplementation strategies in humans. In fact we demonstrated that the topical application of bFGF eye-drops [20] or a cysteine supplementation [21] is safe and effective to accelerate also human epithelial healing after PRK.

The present prospective study was performed to investigate the long-term effects of a l-cysteine oral supplementation in combination to bFGF eye-drops during wound healing process after PRK surgery in myopic patients, and their beneficial effect on the onset of the corneal haze.

## 2. Results

This study investigated the long-term effects of two treatments: patients of group 1 received bFGF for one week, whereas patients of group 2 were supplemented with l-cysteine before and together with bFGF. The supplementation with cysteine was started before surgery in order to adjust the individual levels of cysteine to the highest possible intratissue concentration and to evaluate its postoperatory efficacy evenly in all patients. The study analyzed the recovery from PRK up to 12 months after surgery. There were no significant differences in preoperative variables between the two groups (Table 1). No surgical complications occurred in all the patients involved in our prospective study and no patient developed corneal edema. Moreover no side-effects related to the treatments were reported during the study and none of the subjects were lost to follow-up during the 12-month period.

As a first observation, we were able to detect the significant differences in the postoperative variables between the two groups. In fact we ascertained that the corneal re-epithelization was significantly achieved at day 4 ± 0.5 after the PRK in all the eyes of the patients who were administered oral l-cysteine supplementation in addition to topical bFGF (Group 2), whereas all eyes of the patients who received only bFGF (Group 1) reached the same result in 5 ± 0.2 days (*p* < 0.0001). These data ed the results obtained in a previous study confirm [22] reinforcing the validity of the supplementation of l-cysteine in shortening the healing time of the corneal epithelium after the PRK surgery, and laid the basis for the long-term analysis carried out in this study.

**Figure 1:**
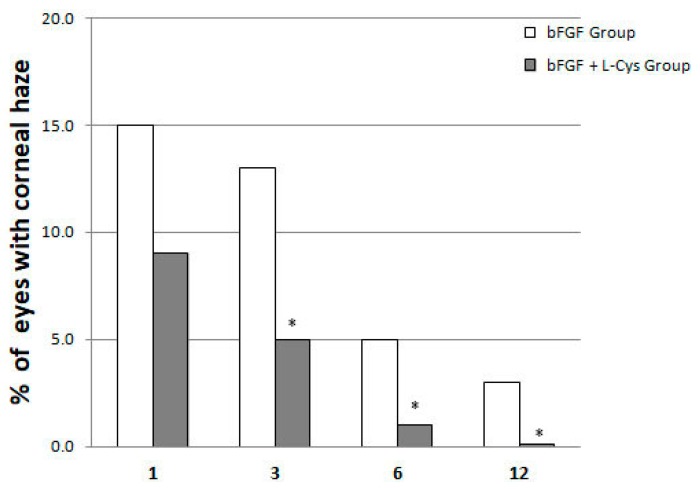
Corneal haze incidence at 3, 6, and 12 month of follow-up as a function of its time of regression in patients who received only bFGF (bFGF Group) and in patients who were administered topical bFGF plus oral l-cysteine supplementation (bFGF + l-cys Group). * *p* < 0.05 bFGF + l-cys Group vs. bFGF Group at each time of follow-up.

The investigation covered the period between one month and twelve months after surgery, in which we evaluated the impact of the beneficial effects of the cotreatment on the later full recovery of vision, in terms of reduction of corneal haze, which is one of the most common postoperative complications. In regard to the occurrence and grading scale of corneal haze, at one month of follow up, 15% and 9% of the eyes of patients belonging to Group 1 and 2, respectively, presented, visually, a mild level of corneal haze (Figure 1). At three months after treatment, trace of corneal haze was observed in 13% and 5% of the eyes of patients belonging to Group 1 and 2, respectively, whereas at six months 5% and 1% showed insignificant haze, and within 12 months the corneal haze has regressed almost completely in Group 2 (Figure 1). Taken together, this data showed that the patients who were postoperatively administered topical bFGF plus oral l-cysteine supplementation (Group 2) developed the corneal haze in a smaller percentage compared to patients who received only bFGF (Group 1). The difference was maintained throughout the follow up, from one month (mild haze) to the end of the study (12 months, full recovery of the Group 2) and it was significant starting from three months after PRK surgery. Moreover, no patient has developed corneal opacification, according to corneal opacification grading [23], in the following months.

**Figure 2:**
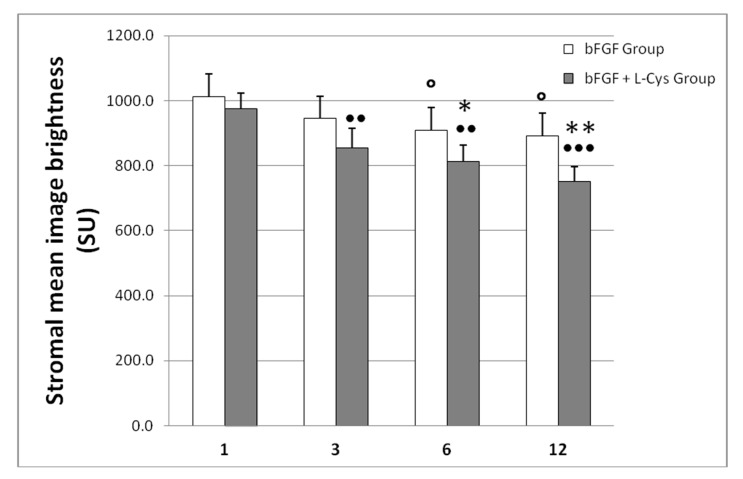
Mean image brightness of the stroma (marker of altered cornea), measured with the ConfoScan 4 confocal microscope and expressed as mean ± SD scatter units (SU) in corneas of patients who received only bFGF (bFGF Group) and of patients who were administered topical bFGF plus oral l-cysteine supplementation (bFGF + l-cys Group). ○ *p* < 0.05 bFGF Group at 3, 6, and 12 months of follow-up vs. bFGF at 1 month of follow-up; ●● *p* < 0.01 and ●●● *p* < 0.001 bFGF + l-cys Group at 3, 6, and 12 month of follow-up vs. bFGF + l-cys Group at 1 month of follow-up; * *p* < 0.03 and ** *p* < 0.001 bFGF + l-cys Group vs. bFGF Group at each time of follow-up.

**Figure 3:**
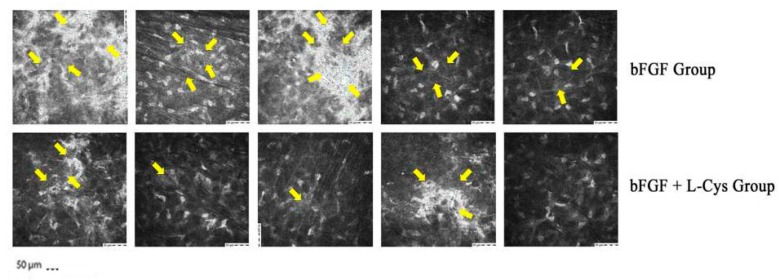
Representative ConfoScan 4 confocal microscope images of the stroma of 5 patients who received only bFGF (bFGF Group) and of 5 patients who were administered topical with bFGF plus oral l-cysteine supplementation (bFGF + l-cys Group). Images were taken at 6 months of follow-up. Yellow arrows indicate the light scattering regions of cornea.

To further evaluate this macroscopic clinical evidence, the haze prevalence was visualized both by slit lamp and confocal microscopy. Confocal microscopy has higher spatial resolution enabling more accurate determination of the structures that contribute to haze [24]. Using confocal microscopy we measured the light scatter of the cornea, which is increased when the architecture of the tissue is compromised. The results of this analysis are shown in Figure 2, whereas representative confocal images taken at 6 months of follow-up are shown in Figure 3. We observed that, if compared to one month after treatment, the bFGF alone had slightly reduced the image brightness after 6 and 12 months from the treatment (*p* < 0.05), whereas the combination of bFGF plus l-cys was effective already at 3 months after the treatment (*p* < 0.01). At the longest times the decrease of brightness generated by cotreatment was more evident than the decrease achieved by bFGF alone; the difference was significant already at 6 months and even more pronounced at 12 months after the treatment (*p* < 0.001; Figure 2). Moreover, comparing the effects of the two different treatments at each time of follow-up, we observed that at one month after treatment there were no statistical differences in the stromal mean image brightness (Figure 2) between the patients of the bFGF Group and the patients of the bFGF + l-cys Group. At 6 months of follow-up, the stromal mean image brightness of the bFGF + l-cys Group was significantly lower than that of the bFGF Group (*p* < 0.03), and, interestingly, it was even lower at 12 month from the treatment (*p* < 0.001). These data demonstrate that post refractive patients can benefit from an oral preadministration of l-cysteine, which triggers both a faster acquisition of the clarity of the healed cornea and a reduction of a complication of the refractive laser surgery. Indeed a higher percentage of patients treated with the l-cysteine supplementation had a significantly improved vision already after 3 months, acquiring a visual recovery already within 6 months of bFGF + l-cys treatment, probably as a consequence of an early stromal epithelial healing.

Furthermore, final mean diopters in spherical equivalent refraction was -0.08 ± 0.3 D in bFGF Group and −0.06 ± 0.2 D in bFGF + l-cys Group (*p* > 0.05), as shown in Table 1.

Next to the complete regressed corneal haze in patients belonging to the bFGF + l-cys Group, final UDVA was equal or superior to 20/25 in 100% of the examined eyes that underwent PRK, without any statistically significant difference in UDVA between the groups (*p* = 0.08). Moreover, no loss of lines of the BCVA was found in both groups at the end of the 12-months follow up.

## 3. Discussion

Despite the success of laser refractive surgery to correct low to moderate order aberrations, the incidence of post operative symptoms remains relatively high, reducing the life quality of patients [25]. In fact, discomfort or pain, delayed epithelial healing, loss of best corrected visual acuity, diffuse haze, and unpredictable refractive complications are unavoidable disadvantages associated with refractive surgery [26]. For these reasons both PRK and the post operative pharmacological management are still evolving [27]. The prompt restoration of the epithelial surface after the surgical procedure is mandatory to both assure and maintain the corneal optical properties; indeed, the persistent blurred vision is related, at the beginning, to an epithelial damage [28] and then to a stromal defect. Corneal stromal wound healing is a very important clinical issue due to great popularity of refractive corneal surgery. It is a very complex and orderly process with keratocyte death and repopulation, sequential transformation of keratocytes into fibroblasts and myofibroblasts, infiltration of limbal and circulating immune cells, and remodeling of the corneal extracellular matrix (ECM) structure [25]. Moreover, it is of fundamental importance that corneal epithelium and stroma interact during healing [3].

Our previous data showed that topical bFGF and oral l-cysteine, alone [20,21] or in combination [22], were able to improve human corneal re-epithelization after the PRK surgery compared to untreated or to bFGF treatment, respectively. However, these studies did not test the effects of the treatments on the long term consequences of the accelerated re-epithelization, for example on the onset and/or the regression of corneal opacity. Indeed, the earlier corneal epithelial complete healing is achieved, the best refractive results after PRK can be expected [1]. The novelty of this study relies on the evaluation of the late beneficial effects of the improved epithelial reconstruction. Moreover the data collected in this study by ophthalmologic examination were validated by the modern technology of confocal microscopy. The reported evidences show that post refractive patients can benefit from l-cysteine oral supplementation in addition to bFGF eye-drops, because the combined treatment plays a fundamental role in the acquisition of the clarity of the healed cornea and minimizes the refractive laser surgery complication.

The activity of l-cysteine can be ascribed to several molecular mechanisms. First of all, l-cysteine acts as a reducing agent, and by enhancing the production of glutathione (GSH) it exerts an important protective antioxidant effect [28].

In rabbit models, the presence of oxygen free radicals after excimer laser ablation has been documented in many studies [29,30,31,32], assuming that reactive oxygen species (ROS) may injure the corneal tissues by decreasing the corneal antioxidant system and/or degrading corneal stromal macromolecules among which proteoglycans and collagen [32], either directly by scission of covalent bonds or indirectly by enhancing their susceptibility to hydrolytic enzymes [11]. This type of acute injury is responsible in part for functional impairment, damage and inflammation [33], and in part for corneal haze because of keratocytes apoptosis and replacement by repopulated and activated keratocytes [34].

Indeed, the use of free radical scavengers, such as ubiquinone Q10 [35] or vitamin E [13], are effective in preventing corneal keratocyte apoptosis and, also, in stimulating corneal re-epithelization. Notably, the immediate post operative use of ascorbic acid significantly reduced lipid peroxidation oxygen radical-mediated tissue damage [11], suggesting that topical ascorbic acid could be considered a complementary treatment in the pharmacological modulation after excimer laser corneal surgery [13]. In this scenario, it is plausible that l-cysteine supplementation exhibits beneficial effects on corneal integrity by preventing an excessive oxidative state due to PRK surgery and thus by reducing the epithelial prolonged inflammation it favors a faster stromal recovery. In fact, it is well established the role of cysteine in alleviating intestinal inflammation and oxidative stress through the inhibition the NF-κB and the activation of the Nrf2 signaling pathways [36], which modulates the proliferation of epithelial cell. Similarly, it is well known that the activation of NF-kB pathway is involved in corneal inflammation [37], which could justify the beneficial effects of l-cysteine on corneal regeneration.

Not of lesser importance, l-cysteine may prevent corneal haze through modulating ECM deposition. Although in most cases of PRK the patients achieve a better vision and the healing process does not present complications, few reports describe a persistent corneal opacity due to stromal haze, probably the result of excessive stromal matrix accumulated during healing and/or persistence of residual matrix-secreting cells [3]. On one hand l-cysteine is essential for a correct synthesis of collagen and proteoglycans. In fact the amino acid plays a fundamental role in the carboxyl-terminal assembly of collagen pro-peptide promoting the formation of structurally sound fibrils and tissues [38]. Moreover, the N-terminal Cys-rich domain is functionally important in stabilizing of the decorin, an extracellular proteoglycan [39].

On the other hand, l-cysteine can reduce the excessive collagen synthesis, as reported in pancreatic fibrosis, by several mechanisms, such as the down-regulation of the pro-fibrotic cytokine transforming growth factor-β (TGF-β) [40]. The occurrence of myofibroblasts and haze formation in the cornea have been attributed to TGF-β hyperactivity released from corneal epithelium following injury to the eye [2]. Recently, the successfully overexpression of decorin, a natural inhibitor of TGF-β, has been demonstrated to suppress fibrosis and myofibroblast formation induced by TGF-β and to decrease α-SMA expression in cultured stromal fibroblasts by over 80% [41]. The involvement of the decorin in our model warrants further investigations.

Taken together, these mechanisms could explain the beneficial effect of l-cysteine exerted on matrix remodeling that would prevent corneal haze. The role and effectiveness of supplements/drugs containing l-cysteine [42] are still unsettled issues, and the present investigation brings the evidences of another benefit of the utilization of l-cysteine in vivo, alongside its low cost and the lack of detectable adverse effect in its short-term treatment. However, despite our encouraging results, the relatively small sample size and the high cost of the bFGF treatment could represent the limitations of the present study.

Moreover, although in previous works the early effects of bFGF and cysteine were tested alone [20,21] or together [22], a study comprehensive of all combinations is necessary to compare their long-term effects; this investigation will strengthen the long-term benefits of cysteine treatment. Although further trials are needed, the results presented in this study underline the importance of cysteine supplementation and bFGF in reducing the corneal haze after photorefractive keratectomy in myopic patients already after 3 months and up to one year. Based on our evidences, cysteine might be a valuable component of an integrated protocol for patients who could benefit of a quicker corneal transparency for restoring the corneal function and, thus, a proper visual quality with a higher satisfaction in the months immediately after the surgery. However, despite our encouraging results, the relatively small sample size and the high cost of the bFGF treatment could represent the limitations of the present study.

In summary, the administration of l-cysteine before the surgery and in association with bFGF in the early postoperative period may represent a good therapeutic strategy to accelerate corneal re-epithelization and to prevent corneal haze in the long-term recovery.

## 4. Materials and Methods

### 4.1. Patients

The study was carried out in accordance with the Declaration of Helsinki for medical research involving human subjects and was authorized by the local Ethical Committee (n. 43/19-05/04/2019). Signed and written informed consent was obtained from all patients accepting to be included in this study.

Only patients affected by myopia, which encountered the following criteria were enrolled: mean spherical equivalent (SE) between −3 and −6 D, best corrected visual acuity (BCVA) equal or greater than 20/25, corneal central thickness (CCT) not less than 540 micron to have a standard corneal profile, and attendance in all follow-up visits. The maximum of treated astigmatism was 0.5. All subjects affected by progressive or unstable myopia, keratoconus, history of herpetic keratitis, previous intraocular and/or corneal surgery, glaucoma, macular and vitreous–retinal disorders, retinal vasculopathy, pregnancy, atopic disease, diabetes, and autoimmune disorders, which could interfere with corneal healing process, were excluded from the present study.

Eighty eyes of 40 otherwise healthy patients (23 males and 17 females) were subjected to bilateral consented PRK for low-medium myopia (mean = −4.5 ± 1.5 D), using the Schwind Esiris Excimer laser (Schwind Eye-Tech Solutions, GmbH, Kleinostheim, Germany). All procedures were performed by one surgeon at the same surgical center, using the same settings.

For this prospective randomized study the enrolled forty patients were divided into two groups: Group 1 included 20 patients (11 males and 9 females, 34.09 ± 8 years of age) who received only topical basic fibroblast growth factor (bFGF, Prodotti Gianni, Milano, Italy; 10 μg/10 μL) four times a day for 7 days starting from the day of surgery in addition to the postoperative standard therapy, which consisted in local tobramycin 0.3%, dexamethasone 0.1% (Tobradex, Alcon Laboratories, Fort Worth, TX, USA), diclofenac eye drops (Voltaren-ofta 0.1%; Novartis, Italy), and 0.2% hyaluronate (Hyalistil, Sifi Spa, Catania, Italy). Group 2 included 20 patients (12 males and 8 females, 37.35 ± 11.5 years of age) who were administered oral l-cysteine supplementation (500 mg/capsule, l-cysteine Natural Point S.r.l., Italy) once a day for 15 days, starting 7 days before PRK, in addition to bFGF eye-drops (10 μg/10 μL) four times a day for 7 days starting from the day of surgery together with the standard therapy. The 8 mm standard treatment was used. Patients were not blinded to pharmaceutical treatments and they were instructed to report immediately for examination if they noticed any decline in vision or other ocular problems.

### 4.2. Ophthalmologic Examinations

All patients underwent a baseline ophthalmological evaluation before laser PRK, subsequently, daily until the complete epithelium healing, and then followed up at 1 month, 3, 6, and 12 months after surgery.

During the clinical visits, best corrected visual acuity (BCVA), Goldmann applanation tonometry, corneal pachymetry, topography and retinal examination, and biomicroscopic observation were assessed. Patients discontinued contact lenses 30 days before surgery.

Beginning on the day after surgery, all subjects were evaluated daily until corneal healing appeared complete by biomicroscopy, and until corneal central thickness was increased according to pachymetry [22].

During the follow-up visits the corneal transparency was evaluated using both slit lamp and confocal microscopy ConfoScan 4 (Nidek Technologies, Fremont, CA) by the same blinded physician. The use of a confocal microscope for measuring the haze is a current, high-resolution measurement, non-invasive, reproducible monitoring technology [43,44] that allows clinicians to quantitative evaluate the haze through the measurements of image brightness of the scattered and reflected light from the cornea, and to follow progression or regression of haze in patients [24,45]. The transparency of the cornea is related to its highly organized structure, and when this complex configuration becomes altered, with penetrating keratoplasty or after corneal refractive surgery, light scatter is increased [46]. To assess corneal stromal density and to evaluate corneal haze in myopic eyes subjected to PRK we referred to both the Heitzmann scale [47] and the Fantes scale [48] for slit lamp measurements and to the McLaren and Bourne standardization for confocal microscopy images [24,45]. Amco Clear, a nontoxic, suspended polymer with particle sizes less than 1 μm, was used as a scatter standard. Amco Clear is used to standardize measurements of scatter and turbidity of liquid suspensions. Its stability (shelf life of 1 year) and uniform light-scattering characteristics make it well suited as a brightness standard for confocal microscopy [24].

Scheimpflug camera was used in the imaging of the corneas by a Pentacam device (Industria Terapeutica Splendore, Napoli, Italy) as this system has the ability to measure the dispersion of light along the optical axis, allowing the detection of changes in the transparency of the lens over time [49].

The tear function was measured by the Schimer tear test [21] without corneal anesthesia, and by the tear film breakup time (BUT).

### 4.3. Statistical Analysis

Data were presented as mean ± standard deviation (SD). As the image brightness values are very similar in both the eyes of each patient, statistical analysis was performed considering the mean of the brightness data of the two eyes for each patient.

The results were checked for normal distribution and analyzed by chi-squared analysis and by one-way analysis of variance (ANOVA) followed by Tukey’s test. The *t*-test applied on two unpaired samples was carried out to compare Group 2 vs. Group 1 at each time of follow-up. Statistical significance level was set at *p* = 0.05. Statistical analysis was performed using the MedCalc 11.4.1.0 Software (Ostend, Belgium).

## Figures and Tables

**Table 1:** A descriptive table of variables by groups. *p* > 0.05 (n.s.) Group 2 vs. Group 1 before and after treatments.

Variables	bFGF Group (Group 1) before Treatment *n* = 20	bFGF + l-cys Group (Group 2) before Treatment *n* = 20	bFGF Group (Group 1) after 1 Month Treatment *n* = 20	bFGF + l-cys Group (Group 2) after 1 Month Treatment *n* = 20	
Gender					n.s.
Male	11	12	11	12	
Female	9	8	9	8	
Age (years)	34.09 ± 8	37.35 ± 11.5	34.09 ± 8	37.35 ± 11.5	n.s.
Nationality	Italian	Italian	Italian	Italian	n.s.
Education	0	0	0	0	n.s.
Primary	0	0	0	0	
Secondary	13	10	13	10	
Higher	7	10	7	10	
Spherical Equivalent	4.00 ± 1.50 D	5.00 ± 1.50 D	−0.08 ± 0.3 D	−0.06 ± 0.2 D	n.s.
BVCA	20/20 ± 5	20/20 ± 5	20/16 ± 4	20/16 ± 4	n.s.

## References

[1] Tomás-Juan J., Larrañaga A.M.-G., Hanneken L. (2014). Corneal Regeneration after Photorefractive Keratectomy: A Review. J. Optom..

[2] Imanishi J., Kamiyama K., Iguchi I., Kita M., Sotozono C., Kinoshita S. (2000). Growth factors: Importance in wound healing and maintenance of transparency of the cornea. Prog. Retin. Eye Res..

[3] Ljubimov A., Saghizadeh M. (2015). Progress in corneal wound healing. Prog. Retin. Eye Res..

[4] Netto M.V., Mohan R.R., Ambrósio R., Hutcheon A.E., Zieske J.D., Wilson S.E. (2005). Wound healing in the cornea: A review of refractive surgery complications and new prospects for therapy. Cornea.

[5] Fagerholm P. (2000). Wound healing after photorefractive keratectomy. J. Cataract. Refract. Surg..

[6] Spadea L., Giammaria D., Trabucco P. (2015). Corneal wound healing after laser vision correction. Br. J. Ophthalmol..

[7] Baldwin H.C., Marshall J. (2002). Growth factors in corneal wound healing following refractive surgery: A review. Acta Ophthalmol. Scand..

[8] Wilson S.E., Mohan R.R., Ambrósio R., Hong J., Lee J. (2001). The corneal wound healing response: Cytokine-mediated interaction of the epithelium, stroma, and inflammatory cells. Prog. Retin. Eye Res..

[9] Suzuki K., Jun S., Ryoji Y., Naoyuki Y., Tai-ichiro C., Keisuke S., Teruo N. (2003). Cell-matrix and cell-cell interactions during corneal epithelial wound healing. Prog. Retin. Eye Res..

[10] Anitua E., Muruzabal F.J., Alcalde I., Merayo-Lloves J., Orive G. (2013). Plasma rich in growth factors (PRGF-Endoret) stimulates corneal wound healing and reduces haze formation after PRK surgery. Exp. Eye Res..

[11] Kasetsuwan N., Wu F.M., Hsieh F., Sanchez D., McDonnell P.J. (1999). Effect of topical ascorbic acid on free radical tissue damage and inflammatory cell influx in the cornea after excimer laser corneal surgery. Arch. Ophthalmol..

[12] Esquenazi S. (2005). Topical Combination of NGF and DHA Increases Rabbit Corneal Nerve Regeneration after Photorefractive Keratectomy. Investig. Opthalmol. Vis. Sci..

[13] Bilgihan K., Ozdek S., Ozoǧul C., Gurelik G., Bilgihan A., Hasanreisoǧlu B. (2000). Topical vitamin E and hydrocortisone acetate treatment after photorefractive keratectomy. Eye.

[14] Rieck P., David T., Hartmann C., Renard G., Courtois Y., Pouliquen Y. (1994). Basic fibroblast growth factor modulates corneal wound healing after excimer laser keratomileusis in rabbits. Ger. J. Ophthalmol..

[15] Assouline M., Hutchinson C., Morton K., Mascarelli F., Jeanny J.-C., Fayein N., Pouliquen Y., Courtois Y. (1989). In Vivo Binding of Topically Applied Human bFGF on Rabbit Corneal Epithelial Wound. Growth Factors.

[16] Scorolli L., Meduri A., Morara M., Scalinci S., Meduri R. (2007). Effect of Cytochrome c Peroxidase on the Corneal Epithelial Healing Process after Excimer Laser Photo-Ablation in Transgenic Mice. Eur. Surg. Res..

[17] Meduri A., Scalinci S.Z., Morara M., Ceruti P., Zigiotti G.L., Scorolli L., Grenga P.L. (2008). Effect of Basic Fibroblast Growth Factor in Transgenic Mice: Corneal Epithelial Healing Process after Excimer Laser Photoablation. Ophthalmologica.

[18] Scalinci S.Z., Scorolli L., Meduri A., Grenga P.L., Corradetti G., Metrangolo C. (2011). Effect of basic fibroblast growth factor and cytochrome c peroxidase combination in transgenic mice corneal epithelial healing process after excimer laser photoablation. Clin. Ophthalmol..

[19] Scorolli L., Meduri A., Morara M., Scalinci S., Meduri R., Colombati S., Greco P. (2008). Effect of Cysteine in Transgenic Mice on Healing of Corneal Epithelium after Excimer Laser Photoablation. Ophthalmologica.

[20] Meduri A., Aragona P., Grenga P.L., Roszkowska A.M. (2012). Effect of Basic Fibroblast Growth Factor on Corneal Epithelial Healing After Photorefractive Keratectomy. J. Refract. Surg..

[21] Meduri A., Scorolli L., Ceruti P., Ferreri G., Grenga P.L. (2008). Role of Cysteine in Corneal Wound Healing after Photorefractive Keratectomy. Ophthalmic Res..

[22] Meduri A., Scorolli L., Scalinci S.Z., Grenga P.L., Lupo S., Rechichi M., Meduri E. (2014). Effect of the combination of basic fibroblast growth factor and cysteine on corneal epithelial healing after photorefractive keratectomy in patients affected by myopia. Indian J. Ophthalmol..

[23] Ho Y.-J., Sun C.-C., Chen H.-C. (2018). Cataract surgery in patients with corneal opacities. BMC Ophthalmol..

[24] McLaren J.W., Wacker K., Kane K.M., Patel S.V. (2016). Measuring Corneal Haze by Using Scheimpflug Photography and Confocal Microscopy. Investig. Ophthalmol. Vis. Sci..

[25] Murueta-Goyena A., Cañadas P. (2018). Visual outcomes and management after corneal refractive surgery: A review. J. Optom..

[26] Stein R. (2000). Photorefractive Keratectomy. Int. Ophthalmol. Clin..

[27] Wilson S.E., Marino G.K., Medeiros C.S., Santhiago M.R. (2017). Phototherapeutic Keratectomy: Science and Art. J. Refract. Surg..

[28] King N., Lin H., Suleiman M.-S. (2010). Cysteine protects freshly isolated cardiomyocytes against oxidative stress by stimulating glutathione peroxidase. Mol. Cell. Biochem..

[29] Bilgihan K., Bilgihan A., Akata F., Hasanreisoğlu B., Türközkan N. (1996). Excimer laser corneal surgery and free oxygen radicals. Jpn. J. Ophthalmol..

[30] Shimmura S., Masumizu T., Nakai Y., Urayama K., Shimazaki J., Bissen-Miyajima H., Kohno M., Tsubota K. (1999). Excimer laser-induced hydroxyl radical formation and keratocyte death in vitro. Investig. Ophthalmol. Vis. Sci..

[31] Hayashi S., Ishimoto S.-I., Wu G.-S., Wee W.R., A Rao N., McDonnell P.J. (1997). Oxygen free radical damage in the cornea after excimer laser therapy. Br. J. Ophthalmol..

[32] Yis O., Bilgihan A., Bilgihan K., Yis N., Hasanreisoglu B. (2002). The effect of excimer laser keratectomy on corneal glutathione peroxidase activities and aqueous humor selenium levels in rabbits. Graefes Arch. Clin. Exp. Ophthalmol..

[33] Bilgihan K., Adiguzel U., Sezer C., Yis Ö., Akyol G., Hasanreisoğlu B., Bilgihan A. (2002). Keratocyte apoptosis and corneal antioxidant enzyme activities after refractive corneal surgery. Eye.

[34] Wilson S.E., He Y.-G., Weng J., Li Q., McDowall A.W., Vital M., Chwang E.L. (1996). Epithelial Injury Induces Keratocyte Apoptosis: Hypothesized Role for the Interleukin-1 System in the Modulation of Corneal Tissue Organization and Wound Healing. Exp. Eye Res..

[35] Brancato R., Schiavone N., Siano S., Lapucci A., Papucci L., Donnini M., Formigli L., Orlandini S.Z., Carella G., Carones F. (2000). Prevention of corneal keratocyte apoptosis after argon fluoride excimer laser irradiation with the free radical scavenger ubiquinone Q10. Eur. J. Ophthalmol..

[36] Song Z.H., Tong G., Xiao K., Jiao L.F., Ke Y.L., Hu C. (2016). l-Cysteine protects intestinal integrity, attenuates intestinal inflammation and oxidant stress, and modulates NF-κB and Nrf2 pathways in weaned piglets after LPS challenge. Innate Immun..

[37] Gong J., Guan L., Tian P., Li C., Zhang Y. (2018). Rho Kinase Type 1 (ROCK1) Promotes Lipopolysaccharide-induced Inflammation in Corneal Epithelial Cells by Activating Toll-Like Receptor 4 (TLR4)-Mediated Signaling. Med. Sci. Monit. Int. Med. J. Exp. Clin. Res..

[38] DiChiara A.S., Li R.C., Suen P.H., Hosseini A.S., Taylor R.J., Weickhardt A.F., Malhotra D., McCaslin D.R., Shoulders M.D. (2018). A cysteine-based molecular code informs collagen C-propeptide assembly. Nat. Commun..

[39] Chen S., Birk D.E. (2010). Focus on Molecules: Decorin. Exp. Eye Res..

[40] Yang L., Shen J., He S., Hu G., Shen J., Wang F., Xu L., Dai W., Xiong J., Ni J. (2012). l-Cysteine Administration Attenuates Pancreatic Fibrosis Induced by TNBS in Rats by Inhibiting the Activation of Pancreatic Stellate Cell. PLoS ONE.

[41] Mohan R., Gupta R., Mehan M.K., Cowden J.W., Sinha S. (2010). Decorin transfection suppresses profibrogenic genes and myofibroblast formation in human corneal fibroblasts. Exp. Eye Res..

[42] Plaza N.C., García-Galbis M.R., Martínez-Espinosa R.M. (2018). Effects of the Usage of l-Cysteine (l-Cys) on Human Health. Molecules.

[43] Smith A.G., Kim G., Porzio M., Allen B., Koach M., Mifflin M., Digre K., Keung B.M., Singleton J.R. (2013). Corneal confocal microscopy is efficient, well-tolerated, and reproducible. J. Peripher. Nerv. Syst..

[44] Bilgihan K., Yesilirmak N., Altay Y., Tefon A.B., Ozdemir H.B., Ozdogan S., Kocamaz M.F., Gurelik G. (2019). Evaluation of Long-Term Corneal Morphology After Photorefractive Keratectomy by In Vivo Confocal Microscopy and Specular Microscopy; 20-Year Follow-Up. Eye Contact Lens.

[45] McLaren J.W., Bourne W.M., Patel S.V. (2010). Standardization of corneal haze measurement in confocal microscopy. Investig. Opthalmology Vis. Sci..

[46] Spadea L., Maraone G., Verboschi F., Vingolo E.M., Tognetto D. (2016). Effect of corneal light scatter on vision: A review of the literature. Int. J. Ophthalmol..

[47] Heitzmann J., Binder P.S., Kassar B.S., Nordan L.T. (1993). The Correction of High Myopia Using the Excimer Laser. Arch. Ophthalmol..

[48] Fantes F.E., Hanna K.D., Waring G.O., Pouliquen Y., Thompson K.P., Savoldelli M. (1990). Wound Healing After Excimer Laser Keratomileusis (Photorefractive Keratectomy) in Monkeys. Arch. Ophthalmol..

[49] Faria-Correia F., Júnior R.A. (2016). Clinical applications of the Scheimpflug principle in Ophthalmology. Rev. Bras. Oftalmol..

